# Daily dose evaluation based on corrected CBCTs for breast cancer patients: accuracy of dose and complication risk assessment

**DOI:** 10.1186/s13014-022-02174-4

**Published:** 2022-12-12

**Authors:** Vincent C. Hamming, Sebastian Andersson, John H. Maduro, Johannes A. Langendijk, Stefan Both, Nanna M. Sijtsema

**Affiliations:** 1 Department of Radiation Oncology, University Medical Center Groningen, University of Groningen, Hanzeplein 1, 9713 GZ Groningen, The Netherlands; 2 RaySearch Laboratories, Stockholm, Sweden

**Keywords:** CBCT, CBCT correction, Breast, Dose evaluation, Image quality evaluation

## Abstract

**Objectives:**

The goal of this study is to validate different CBCT correction methods to select the superior method that can be used for dose evaluation in breast cancer patients with large anatomical changes treated with photon irradiation.

**Materials and method:**

Seventy-six breast cancer patients treated with a partial VMAT photon technique (70% conformal, 30% VMAT) were included in this study. All patients showed at least a 5 mm variation (swelling or shrinkage) of the breast on the CBCT compared to the planning-CT (*pCT*) and had a repeat-CT (*rCT*) for dose evaluation acquired within 3 days of this CBCT. The original CBCT was corrected using four methods: (1) HU-override correction (*CBCT*_*HU*_), (2) analytical correction and conversion (*CBCT*_*CC*_), (3) deep learning (DL) correction (*CT*_*DL*_) and (4) virtual correction (*CT*_*V*_). Image quality evaluation consisted of calculating the mean absolute error (*MAE*) and mean error (*ME*) within the *whole breast clinical target volume* (*CTV*) and the field of view of the CBCT minus 2 cm (*CBCT-ROI*) with respect to the rCT. The dose was calculated on all image sets using the clinical treatment plan for dose and gamma passing rate analysis.

**Results:**

The *MAE* of the *CBCT-ROI* was below 66 HU for all corrected CBCTs, except for the *CBCT*_*HU*_ with a *MAE* of 142 HU. No significant dose differences were observed in the *CTV* regions in the *CBCT*_*CC*_, *CT*_*DL*_ and *CT*_*v*_. Only the *CBCT*_*HU*_ deviated significantly *(p* < *0.01)* resulting in 1.7% *(*± *1.1%)* average dose deviation. Gamma passing rates were > 95% for 2%/2 mm for all corrected CBCTs.

**Conclusion:**

The analytical correction and conversion, deep learning correction and virtual correction methods can be applied for an accurate CBCT correction that can be used for dose evaluation during the course of photon radiotherapy of breast cancer patients.

## Introduction

In modern radiotherapy, patient positioning is crucial for an accurate dose delivery during radiotherapy treatments. This can be achieved by verifying and subsequently correcting patient position prior to beam delivery. A current often used treatment position verification imaging method is cone beam computed tomography (CBCT). By comparing patient anatomy to that of a reference acquired at the time of treatment planning, the patient’s position can be optimized prior to treatment delivery. It is possible that anatomical deviations with respect to the reference are visible on the CBCT, e.g. due to weight loss or gain, swelling or differences in patient posture compared to the reference due to stress or anxiety during reference computed tomography (CT) acquisition. It is however unclear what the consequences of these deviations are for the dose distribution. Especially highly modulated plans (e.g. volumetric modulated arc therapy (VMAT) and intensity modulated radiotherapy) are more susceptible to anatomical deviations. Hence, it is unknown whether the original treatment plan still suffices for the new anatomy. Ideally the CBCT could be used for dose evaluation and thereby verifying if the treatment plan still suffices or needs to be updated. However, the CBCT often has a small field of view (FOV) and includes low frequency artifacts reducing the quality of the CBCT. Most importantly, the relative large contribution of scattered X-rays in CBCTs results in an inaccurate representation of the Hounsfield units (HU), causing an uncertainty in the HU-to-electron density conversion resulting in uncertainty in the final calculated dose distribution [1–7]. To accurately assess the dosimetric consequences of anatomical deviations found on CBCT a repeat CT is generally acquired. In many cases dose evaluation on these repeat CTs does not show dose deviations that need a plan adaptation. A method for reliable dose evaluation based on CBCTs would reduce the number of unnecessary repeat CTs reducing the imaging dose to patients and the workload for the radiotherapy department.

The goal of this study is to validate different CBCT correction methods to select the superior method that may be used for dose evaluation in breast cancer patients with large anatomical changes treated with photon irradiation to identify the possible need for a plan adaptation. For this purpose, CBCT correction methods have been assessed by quantifying the resulting HU and dose accuracy and their influence on complication risk assessment.

## Methods

### Included patients

76 left or right-sided breast cancer patients undergoing whole breast radiotherapy without nodal involvement were selected retrospectively. 43 patients received a simultaneous integrated boost treatment with a fractionation scheme of 21 F × 2.17 Gy/F(+ 0.49 Gy/F) while the remaining 33 patients received a fractionation scheme of 16 F × 2.66 Gy/F (no boost).

All patients were treated on the Elekta Infinity.™ linear accelerator (Elekta AB, Sweden). CBCTs were acquired (X-ray volumetric imaging (XVI), v5.0.4, Elekta AB, Sweden, 195° arc, 120 kV, 32.5 s, 0.4 mAs) for the first three fractions and thereafter once a week, according to the extended no-action level +  + protocol. [8]

Only patients with physical anatomical surface deformations > 5 mm on the CBCT compared to the planning CT *(pCT)* due to swelling or shrinkage were included in the study. Following the local clinical workflows for patients with deformations > 5 mm, a repeat CT *(rCT)* was acquired within three days after the CBCT showing the deformations to evaluate the dosimetric consequences. The *pCT* and *rCT* were acquired on the Somatom-Definition AS CT-scanner (Siemens, Forchheim, Germany).

The whole breast was visible on the CBCT and no clear artifacts were visible on either the *pCT*, *rCT* or CBCT. All patients were treated with a partial VMAT technique with 70% of the dose delivered with a conformal technique consisting of two tangential fields, and 30% with 4 short VMAT arcs of 40–80°, depending on the anatomy of the patient. In general start-stop gantry angles are in-between 20°and 300° for arcs 1 and 2 and in-between 90°and 150° for arcs 3 and 4 for left sided breast cancer patients (60°–340° and 210°–270° for right sided breast cancer patients).

### CBCT correction methods

Four different CBCT correction methods have been applied.

#### HU-override correction

A standard built-in correction method for imported CBCTs in the treatment planning system (TPS, RayStation Research version 9.10.1, RaySearch, Sweden) is the creation of a patient specific CBCT number to density table. The density table is created by using an automatic multilevel-thresholding, based on algorithms that classify CBCT voxels with similar HUs into several ranges, based on grey levels [9]. Automatic thresholding was applied to obtain a uniform method over all CBCTs. Six different densities are assigned to the CBCT; air (0.00121 g/cm^3^), lung (0.26 g/cm^3^), adipose (0.95 g/cm^3^), tissue (1.05 g/cm^3^), cartilage/bone (1.6 g/cm^3^) and other (3 g/cm^3^). Although densities were assigned, the raw CBCT image remained unaltered. This method is referred to as the HU-override correction method and results in the *CBCT*_*HU*_.

#### Analytical correction and conversion

An early version of this algorithm is available in a research version of the TPS RayStation 9B. The algorithm is based on the work of Marchant et al. and works in an iterative manner, consisting of two main parts [10]. The first part of the algorithm is to find a conversion from the CBCT grey level scale to the *pCT* grey level scale and the second part is to find a correction map that removes low frequency artifacts. In the first part, a joint histogram is created (after a deformable registration) of the *pCT* (reference) and the *CBCT* (target). Tissue pairs can be observed within this histogram which are used to construct a conversion function based on linear interpolation. In a subsequent correction step a difference map was created between the *pCT* and CBCT, which is limited to the FOV of the CBCT. This difference map was filtered with a low pass filter and added to the CBCT to reduce low frequency artifacts. This analytical correction and conversion method results in the CBCT_cc_.

#### Virtual CT

The virtual CT is created by first deforming a reference *CT* to the CBCT, hence, the densities from the *CT* will be used for the virtual CT in most of the image. Any air pocket present in either the *CT* or the CBCT is replaced by the values from the CBCT corrected with the analytical correction and conversion method (CBCT_cc_), yielding the *CT*_*V*_.

#### Deep learning correction

From the 76 patients, randomly 37 patients have been selected and have been prepared to create a deep learning model to correct the CBCT. while the remaining 39 patients were used as an independent test set for the validation of the model. The patient group used for model training consisted of 17 left-sided, 19 right-sided and 1 bilateral breast cancer patient, of which 22 received a SIB treatment. During training of the model the data was split into two groups; (1) training and (2) validation. 31 out of the 37 patients were used for training and the remaining 6 patients were used for validation. Augmentation of the images was performed by applying ±7° rotations and ±7 pixels translations during training. The preparation of patients consisted of creating a deformed *rCT* with the CBCT as reference and the original *rCT* as target, where the deformation was focused on the FOV of the CBCT minus 2 cm. The resulting image was a *rCT* deformed to the CBCT with a FOV equal to that of the CBCT. The *rCT* was used because of superior anatomical similarities with the CBCT compared to the *pCT*. The model used is similar to the cycle-generative adversarial network (CycleGAN) architecture [11]. However, an additional paired data term was used in the cost function during training which was equal to the difference between the deep learning CT and the deformed *rCT* (|*CT*_*DL*_-deformed *rCT*|). The paired weight was set to 3 while the cyclic weight was 7. This correction method results in the *CT*_*DL*_*.*

### Image quality evaluation

A script in RayStation was used to consistently process the remaining 39 patients that were used as an independent test set. Initially all patients had a (deformed) *pCT*, (deformed) *rCT* and HU-override corrected CBCT as image set available in RayStation. Both the *pCT* (original + deformed) and *rCT* (original + deformed) already had CTVs delineated which were unaltered in the workflow described. All the CTVs were contracted to be within 5 mm of the BODY contour on the *rCT* and *pCT*. The following actions were performed in consecutive order within the script:

1. The BODY contour was set as type ‘External’ on the *pCT* and *rCT*
2. A three degree of rotation rigid registration was performed between the *rCT* and the CBCT
3. An additional structure ‘*CBCT-ROI*’ was created on all image sets which is the intersection between the FOV of the CBCT minus 2 cm and the BODY contour and all structures were copied rigidly from the *rCT* to the CBCT.
4. The correction methods were applied to the CBCT resulting in the additional *corrected and converted CBCT (CBCT*_*CC*_*), virtual CT (CT*_*V*_*)* and *deep learning CT (CT*_*DL*_*)*.

By using a rigid registration, equal volumes of the ROIs between the different image sets were ensured. After ROI transfer to the CBCT image sets, all the contours were visually checked to be within the BODY and if necessary, slightly altered. The *rCT* image was used as reference to evaluate the accuracy of HU of the different corrected CBCTs. The HU accuracy was quantified based on the mean absolute error (*MAE*) and mean error (*ME*), defined in Eqs. 1 and 2, for the ROIs ‘*CBCT-ROI*’ and the *whole breast CTV*.

1 $$MAE= \frac{\sum_{i=1}^{n} \left|{HU}_{rCT(i)}-{HU}_{synthetic CT(i)}\right|}{n}$$

2 $$ME= \frac{\sum_{i=1}^{n} {(HU}_{rCT(i)}-{HU}_{synthetic CT\left(i\right)})}{n}$$

In Eqs. 1 and 2$i$ represents the voxel within the ROI to be analyzed and *n* is equal to the number of voxels in the ROI. Statistical analyses were performed using the *rCT* as reference with a two-tailed student’s *t*-test using independent samples with equal variances.

### Dose calculation accuracy

For the purpose of dose calculation accuracy, the previous script was extended. The changes consisted of using a deformable registration between the rCT and the CBCT in step 2 and consequently warping structures to the CBCT in step 3. More steps were added to the script:

(5) All voxels in the BODY contour, but outside the FOV of the CBCT, were set to a density of 1 g/cm^3^
(6) The dose is calculated (Collapsed Cone algorithm) on these corrected CBCTs using the original treatment plan from the *pCT*
(7) Finally the *whole breast CTV*, *boost CTV* and heart dose-volume based parameters (D1, D2, D95, D98, D99 and average) of the *pCT*, *rCT* and corrected CBCTs were exported for data analysis within Excel (Office 365, version 16.0.14131.20326).

Boxplots were created for visualization of the results. Statistical analyses were performed using the *rCT* as reference with a two-tailed student’s *t*-test using independent samples with equal variances. Global 3D gamma passing rates (GPR) were calculated in the MICE toolkit (NONPIMedical AB, Sweden, Umeå, version 2021.2.1) between the *rCT* (reference) and corrected CBCT dose distributions with gamma passing criteria of 2%/2 mm, 3%/3 mm and 5%/5 mm with reference doses of 42.47 Gy or 56.07 Gy depending on the prescription. All voxels with a dose > 0 in the reference dose distribution were taken into account.

The average heart dose was used in combination with the normal tissue complication probabilities (NTCP) model for acute coronary events (ACE) to determine the differences between the image sets. The NTCP model is based on data from Darby et al. and Van Den Bogaard et al. and incorporates age and multiple risk factors to calculate the ACE risk [12, 13]. As this data was unknown for the patient population, the ACE risk was determined by interpolation between the corresponding dose values in > 70 years for the minimum risk, and < 40 years for the maximum risk as is presented by Boersma et al. [14] This model is clinically implemented within the model-based selection for photon vs. proton treatment of breast cancer patients in The Netherlands [14–16].

## Results

### CBCT correction methods

**Fig. 1:**
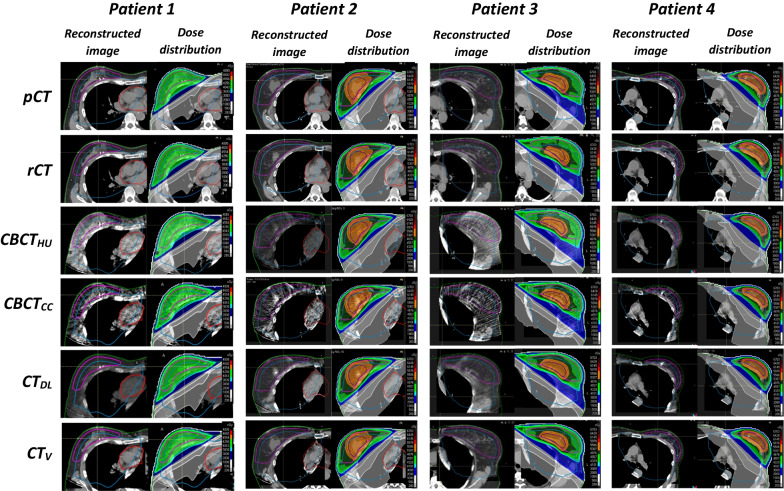
Overview of the different images used during analysis for four different patients. The images show the different ROIs used during analyses, including the calculated dose distribution according to the original treatment plan. The W/L was set to 400/40HU. The ROIs present are External (green), whole breast CTV (pink) boost CTV (pink), heart (red) and CBCT-ROI (blue).The ROIs are warped from the rCT, hence the differences between the image sets

For all patients the CBCT correction was performed without any problems by the script. In Fig. 1 the *pCT*, *rCT* and the four corrected CBCTs are presented for four patients. The training for the deep learning correction based on the paired data |*CT*_*DL*_-deformed *rCT*| continued for 30 epochs. The optimal model was obtained after the 22nd epoch, yielding a mean absolute error (*MAE*) of 64 HU in the validation set.

### Image quality evaluation

For all 39 patients in the test set all CTVs were within the BODY contour after rigid transformation. A slight volume difference existed between the unaltered *whole breast CTV* from the *pCT* and the other image sets. The *CBCT-ROI* is copied rigidly to all image sets to ensure equal volumes for optimal comparison. The *MAE* and *ME* results for the HU accuracy evaluation are presented in Table 1.

**Table 1:** Overview of the HU accuracy evaluation in terms of mean absolute error (MAE) and mean error (ME) in the test set. The results are presented as mean ± 1σ and the range ([min; max]) for the whole breast CTV and CBCT-ROI

Image comparison	Whole breast CTV		CBCT ROI	
	MAE [HU]	ME [HU]	MAE [HU]	ME [HU]
rCT-pCT	15 ± 3 [12;24]	2 ± 4 [−9;9]	32 ± 7 [23;51]	1 ± 8 [−18;23]
rCT- CBCT_HU_	80 ± 25 [35;169]	73 ± 29* [15;167]	142 ± 21 [107;198]	18 ± 42 [−69;130]
rCT- CBCT_CC_	50 ± 13 [29;81]	3 ± 8 [−19;22]	66 ± 9 [50;84]	2 ± 9 [−15;26]
rCT- CT_DL_	35 ± 15 [17;75]	−7 ± 28 [−72;57]	61 ± 13 [42;110]	−18 ± 25 [−79;28]
rCT- CT_V_	19 ± 6 [12;42]	3 ± 5 [−10;15]	48 ± 6 [35;68]	−3 ± 9 [−24;27]

The rCT was used as reference during the image quality evaluation. The asterisks (*) indicate a significant difference (*p* < 0.001) between the rCT and image set

In general the differences were larger for the *CBCT-ROI* because of the varying tissues (e.g. air, muscle and bone) resulting in a larger HU range within this region compared to the *whole breast CTV*. The *CBCT*_*HU*_ shows the largest *MAE* differences compared to the *rCT.* Lower *MAE* values were found for other correction methods. The *ME* of the HU values of the *CBCT*_*HU*_ (*p* < *0.001)* were significantly different from those of the *rCT* for the *whole breast CTV*.

### Dose calculation

The original treatment plan generated on the *pCT* is used to calculate the dose on the corrected CBCTs, see Fig. 1 for the dose distributions. The dose difference relative to the prescribed dose is calculated between the *rCT* (reference) and the different image sets (*pCT, CBCT*_*HU*_*, CBCT*_*CC*_*, CT*_*DL*_*, CT*_*V*_*).* The *rCT* is used as reference because it is most representative for the CBCT.

**Fig. 2:**
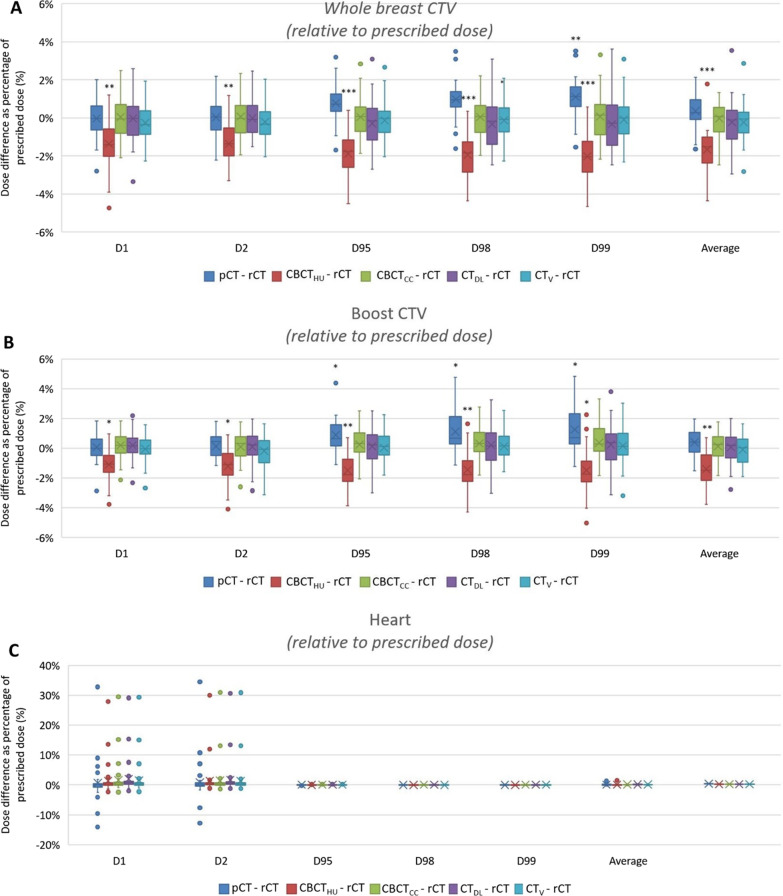
The dose differences relative to the prescribed dose between the different image sets and the repeat CT (rCT) for **A** the whole breast CTV, **B** the boost CTV and **C** the heart. The asterisks indicate a significant difference between the specific image set compared to the rCT. *: *p* < 0.05, **: *P* < 0.01, ***: *P* < 0.001

The results are presented as boxplots in Fig. 2 for the *whole breast CTV* and *heart* which are available for all patients, and the *boost CTV* which is available for twenty-one patients. The asterisks above the graph indicate the level of significance between the *rCT* and the specific image set. The dose difference relative to the prescribed dose between the *rCT* and *pCT* is on average 0.4% *(*± *0.8%)* which is assumed to be due to volume changes present between the *rCT* and *pCT*. It is shown that in both CTV cases the *CBCT*_*HU*_ shows a significant difference compared to the *rCT*, resulting in a systematic underdosage calculated by the *CBCT*_*HU*_. The average dose difference between the *rCT* and *CBCT*_*HU*_ is −1.7% *(*± *1.1%)* for the *whole breast CTV* and −1.4% *(*± *1.2%)* for the *boost CTV*, corresponding to an absolute difference of 73 cGy and 78 cGy respectively. From the four CBCT correction methods, the *CBCT*_*CC*_ shows the smallest average difference with the *rCT*: 0.0%*(*± *1.0%)* and 0.1%*(*± *1.0%)* difference, respectively for the *whole breast CTV* and *boost CTV*, compared to −0.2%*(*± *1.2%)* and 0.1%*(*± *1.2%)* for the *CT*_*DL*_ and −0.2%*(*± *1.0%)* and −0.1%*(*± *1.0%)* for the *CT*_*V*_ respectively.

The global 3D GPR is calculated for the passing criteria 2%/2 mm, 3%/3 mm and 5%/5 mm between the *rCT* dose distribution (reference) and corrected CBCTs. The overview of GPR (mean ± 1σ) is presented in Table 2 for the different criteria.

**Table 2:** The results for the global 3D gamma passing rates between the dose distribution of the rCT (reference) and the corrected CBCTs

	*CBCT*_*HU*_	*CBCT*_*CC*_	*CT*_*DL*_	*CT*_*V*_
Gamma criteria: 2%/2 mm	95.7 ± 2.5%	97.3 ± 1.8%	97.1 ± 2.1%	97.3 ± 1.8%
Gamma criteria: 3%/3 mm	98.0 ± 1.5%	98.7 ± 1.1%	98.7 ± 1.2%	98.8 ± 1.1%
Gamma criteria: 5%/5 mm	99.4 ± 0.6%	99.6 ± 0.6%	99.6 ± 0.6%	99.6 ± 0.6%

The numbers presented are mean ± 1σ of the gamma passing rates for the different passing criteria

In general, the GPR for the CBCT_HU_ are worse than for the other corrected CBCTs, which is in agreement with the results of the dose-volume analysis. The GPR results for the CBCT_CC_, CT_DL_ and CT_V_ are > 97% for 2%/2 mm and the difference between those three methods is always < 0.2% for all gamma criteria.

**Fig. 3:**
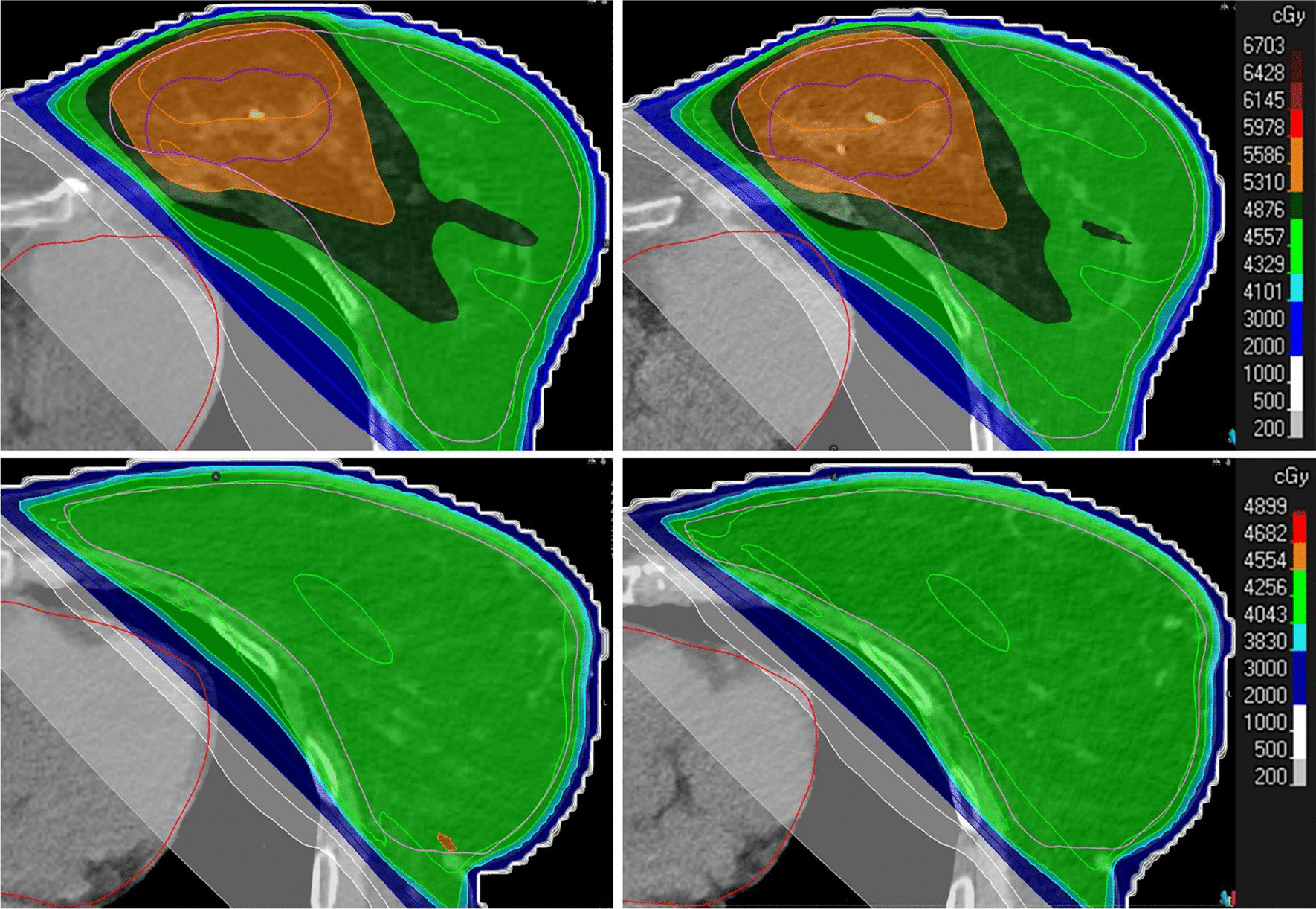
Overview of the pCT (left) and rCT (right) for two patients showing deviations in the D1 and D2 in the heart due to a changed heart position resulting in a higher dose in a small volume of the heart

The heart dose is on average very similar to the *rCT* heart dose, as can be seen in Fig. 2C. The heart dose for all images sets shows no significant differences compared to the *rCT* over all dose statistics. The difference in mean heart dose varied on average between 164 cGy (*rCT*) and 170 cGy (*CT*_*V*_). These differences correspond to a maximum change in risk of an ACE by 0.05%, depending on the age and possible risk factors [14]. However, for specific patients large differences exist specifically for the dose volume parameters *D1* and *D2* as can clearly be seen in Fig. 2C. This is caused by a change in lung volume, which is illustrated in Fig. 3 for two patients. Although the average heart dose varies slightly, the *D1* and *D2* values can show a large variation for individual patients. Figure 3 (bottom) presents a patient with a *D2* dose difference of 1466 cGy between the *pCT* and *rCT*, with a *D2* heart dose difference on corrected CBCTs in the order of 1300 cGy as well. In total 10 patients (7 deep inspiration breathhold (DIBH) and 3 free breathing) showed a difference > 2% for *D1* and *D2* compared to the *pCT* corresponding to an absolute dose difference of at least 85 cGy. 4 of these patients showed > 2% dose deviation on the *D1* or *D2* for the *rCT* while the dose on the synthetic CTs was < 2%. Moreover, all patients showing > 2% difference on any corrected CBCT for the *D1* or *D2*, also showed > 2% difference on the corresponding *rCT*.

## Discussion

Many papers already described the correction of clinical cone-beam CTs for dose evaluation in adaptive radiotherapy [17–21]. However, HU accuracy comparison combined with dose calculation accuracy and complication risk assessment of four different CBCT correction methods available in a research version of a commercial TPS for breast cancer patients treated with photon irradiation has not been published previously. Therefore, the current study provides valuable information for the clinical implementation of using corrected CBCTs for dose evaluation and identifying the need for plan adaptations based on target coverage and dose to OAR in photon breast cancer patients.

Compared to the *rCT* the *CBCT*_*HU*_ shows the largest differences for *MAE* and *ME* which indicates that this correction method is the least accurate correction method. The results for the *CBCT*_*CC*_ are similar to that of the *CT*_*DL*_ and are comparable to the *MAE* of 56 HU determined by Kidar et al. [22] Shi et al., Marchant et al. and Niu et al. already showed that by using the analytical correction and conversion the HU accuracy improved significantly [10, 23, 24]. The HU accuracy in the *CT*_*DL*_ in this study is superior to the research of Maspero et al. where a *MAE* of 66 HU was calculated but is comparable to the study of Wang et al. where a *MAE* of 39 HU is determined within the breast region for CycleGAN deep learning corrected CBCT specific for breast patients. [21, 25] Similar deep learning corrected CBCTs image quality research is performed for head and neck (H&N), thorax and abdominal regions. Ranges of resulting *MAE*s in literature are found to be 19–77 HU (H&N), 47–94 HU (thorax) and 42–87 HU (abdomen) respectively [18–21, 26–29] It is clear that for every site a (wide) range in *MAE* is observed. General reasons to take into account causing variations in *MAE* are (1) time difference between CBCT and *rCT* acquisition, (2) use of immobilization techniques and (3) the ROI which is used for *MAE* determination. Decreasing the time between the *rCT* and CBCT acquisition and applying immobilization on both the CBCT and *rCT* results in optimal anatomical similarities, which is expected to result in a decrease in *MAE* between the *rCT* and *CT*_*DL*_.[30] The size of the ROI for *MAE* calculation determines the variety of tissues included and thereby increases the range of HU included. This will consequently increase the *MAE* between images.

Considering the accuracy of dose calculation, different dose-volume parameters were analyzed for the *whole breast CTV* and the *boost CTV*. Due to volume changes visible on the *rCT*, a dose difference is expected between the *rCT* and *pCT*. In this research, this difference is determined to be 0.4% *(*± *0.8%)* for the *whole breast CTV*. Because the *whole breast CTV* is delineated by the physician on both the *pCT* and *rCT* prior to this study, the dose difference is assumed to be solely due to the volume changes. As the CBCTs used for the CBCT correction methods are always acquired maximum three days before the *rCT* it is assumed that the patient posture and anatomy of the CBCT corresponds better to the *rCT* than to the *pCT*.

The *CBCT*_*HU*_ shows significant dose differences for all dose statistics, resulting in an average systematic underdosage of 1.7% *(*± *1.1%)* for the *whole breast CTV*. This is also observed by Dunlop et al., who showed that the auto segmentation in RayStation underestimates the proportion of lower-density tissues, corresponding to an underestimation of the dose for the CBCT [31]. Using a patient specific HU correction method results in general in a dose deviation of 1%-2% [6, 31–34]. It should be noted that for these literature studies a much smaller population of 10–11 patients was used, while in the current study 39 patients were used to evaluate the dose. The analytical conversion and correction results in 0.0% *(*± *1.0%)* and 0.1% *(*± *1.0%)* dose difference compared to the *rCT* for the *whole breast CTV* and *boost CTV* respectively. Marchant et al. used a similar correction for 15 lung patients and calculated a mean dose difference of 0.5% *(*± *0.7%)* which was similar to the dose difference calculated in the current study. [35] A similar but inferior CBCT correction method more frequently used in literature is the histogram matching correction method, which excludes low frequency artifact correction. Onozato et al. and Abe et al. calculated a dose difference of 0.8% and 2% respectively with the histogram matching correction. [36, 37] Only three other studies performed voxel-based photon dose calculation on *CT*_*DL*_, resulting in dose differences of 0.1–1% which is similar to the −0.2% *(*± *1.2%)* determined in our study [21, 38, 39]. The *CT*_*V*_ as used within the current study has not been reported in literature. It should however be mentioned that for the purpose of breast patients, the additional corrections of the *CT*_*V*_ are negligible. Therefore, this CBCT correction method should be further investigated in target regions with air-pockets present. From the corrected CBCTs, only the *CBCT*_*HU*_ is significantly different compared to the *rCT* hence, the other three correction methods are accurate to use for dose evaluation and identifying patients with a need for a plan adaptation based on increased dose to OAR or reduced target coverage. This could potentially replace the *rCT* which remains currently necessary for the evaluation of the dosimetric consequences of anatomical deviations. Consequently, less unnecessary *rCTs* will be acquired resulting in lower imaging dose to the patient and a decreased workload for the radiotherapy department.

The global 3D gamma passing rate (GPR) was better than 95% for the strictest criteria of 2%/2 mm for all corrected CBCTs and even better than 97% when excluding the *CBCT*_*HU*_. This agrees well, or is even better than described in other studies. For HU-override correction, or multi-level thresholding correction, the GPR published on patient studies are limited. Giacometti et al. and Onozato et al. determined a GPR > 95% for multiple anatomical sites with respectively 2%/0.1 mm and 1%/1 mm as passing criteria [36, 40]. GPR values for the currently used analytical correction and conversion method are not published in literature for photon dose calculation in breast patients. However, multiple articles have used the histogram matching correction method in combination with GPR analysis. For multiple GPR criteria it is found that the GPR is between 94%-100% including multiple sites. [6, 36, 41, 42] Regarding GPR calculations on CycleGAN deep learning corrected CBCT, two studies can be identified for photon dose calculations specific for breast patients. [21, 25] Maspero et al. and Wang et al. included a 2%/2 mm criteria and calculated a GPR of 89% *(*± *9%)* and 84% *(*± *4%)* respectively. The GPR of 97% *(*± *2%)* determined in this research shows superior accuracy of dose calculation on deep learning corrected CBCTs. Five other studies can be identified for other anatomical sites, all including a 2%/2 mm criteria, showing GPR ranging from 92 to 99.5%. [29, 39, 43–45] It should be noted that GPR results are difficult to compare, as frequently the passing criteria and dose threshold levels are different or not mentioned.

The complication risk assessment for acute coronary events (ACE) is based on the mean heart dose (MHD). In this study it is determined that the difference of absolute average MHD between all image sets is 6 cGy *[164 (rCT); 170 cGy (CT*_*V*_*)]*. This corresponds to a maximum change in risk of an ACE by 0.05%, depending on the age and possible risk factors [14]. Although the delineation of the heart is partly outside the FOV of the CBCT, hence, a density of 1 g/cm^3^ is assigned, this did not influence the results for the MHD. Although the MHD is very similar between the image sets, the *D1* and *D2* results can vary significantly on individual bases as shown in Fig. 3. However, this result is not due to the correction method, but due to a different heart position between the *rCT* and *pCT*. Here it is shown that when a *D1* or *D2* dose deviation > 2% is determined for any corrected CBCT compared to the *pCT*, this was also always the case for the corresponding *rCT*. Conversely, in 4 out of the 10 cases a > 2% dose deviation on the *rCT* did not correspond to a > 2% dose deviation on the corrected CBCTs. No clear difference was observed between DIBH and free breathing patients, indicating that a change in lung volume is not limited to either one of the groups. Differences in lung volume, and thereby a varying distance between the heart and thoracic wall, is most likely the reason for the change in heart dose, which can be caused by anxiety or stress during CT acquisition. Hence, the *rCT* is not always an accurate representation of the position and can therefore result in suboptimal clinical decisions. The corrected CBCT therefore provides valuable information and shows great promise as an indicative tool as the clinical treatment position is actually taken into account. It should also be mentioned that Van Den Bogaard et al. determined that the volume of the left ventricle receiving 5 Gy and the mean dose to atherosclerotic plaque within the left anterior descending coronary artery (LAD) is a superior predictor for ACE. [13, 46] In the case of left-sided breast treatment, the small region of higher dose might include the left ventricle or the LAD. As a consequence, the difference in dose to the left ventricle might vary severely, resulting in a higher predicted ACE risk, while the MHD only varies slightly. Further work is required to accurately assess the possible increased ACE risk by taking the dosimetric changes to the left ventricle and LAD into account.

A limitation of this study is that the registration performed for the dose evaluation in RayStation differs from the clinical procedure during CBCT evaluation. The rigid registration in RayStation is a three degrees of freedom grey level based registration, focused on the bone structures within the thoracic wall. The clinical automatic registration in XVI is based on the thoracic wall, where after, if applicable, it is checked whether the surgical clips marking the surgical cavity are within 5 mm of their *pCT* position. As a result, the deviation between the clinical and RayStation registration might differ up to a few millimeters which was determined retrospectively. The effect of this difference is estimated to be negligible within the aims of this study. However, it should be taken into account when implementing this within a clinical procedure. A second limitation is the FOV of the CBCT, which in general does not encompass the whole patient but only relevant anatomy for position verification purposes. A correction is therefore necessary for areas which are being traversed by beams outside the FOV. Ideally this is solved by copying densities from the reference CT to these areas outside the FOV [31, 47]. However, it was not possible to use this principle due to lack of functionality in the training of the deep learning model. Therefore, in this study it is solved by assigning a density of 1 g/cm^3^. Moreover, all the beams from the original *pCT* treatment plan, tangential conformal fields and small arcs, enter mainly through the FOV of the CBCT. A very limited amount of dose is delivered through the area of an assigned density of 1 g/cm^3^, which otherwise would also be very similar to this density. It is therefore assumed that within the scope of this study this effect is negligible. However, for other treatment sites using e.g. full arcs, this effect becomes more important and cannot accurately be solved by assigning a density of 1 g/cm^3^. The third and last limitation is the planning technique used in this study. All patients received a partial VMAT treatment, with a dose contribution of 70% from conformal beams and 30% from VMAT beams. All results within this study are based on this planning technique, while by changing the ratio of conformal to VMAT or by introducing intensity-modulated radiation therapy (IMRT) the complexity of the plan differs. As the dose assessment is affected by the complexity, the dose assessment of the corrected CBCTs might be different for different planning strategies. However, we expect that these effects are limited and would not influence the conclusions of this study.

## Conclusion

The analytical correction and conversion, deep learning correction and virtual correction methods can be applied for an accurate CBCT correction that can be used for dose evaluation during the course of photon radiotherapy of breast cancer patients. The usage of corrected CBCTs therefore shows great promise in clinical implementation to determine whether the anatomical deviations result in clinical unacceptable dose deviations based on dose to OAR and target volumes that need a plan adaptation.

## Data Availability

All the obtained data used for data analysis within this study is available through Mendeley Datasets: Hamming, Vincent (2022), “Dose calculation on the CBCT”, Mendeley Data, V1, https://doi.org/10.17632/b3dcthxfkt.1. The data is separated into a file with the dose statistics, the HU-values and the gamma passing rates.

## Abbreviations

ACE: Acute coronary event
CBCT: Cone beam computed tomography
CTV: Clinical tumor volume
CycleGAN: Cycle-generative adversarial network
DIBH: Deep inspiration breathhold
DL: Deep learning
FOV: Field of view
GPR: Gamma passing rate
HU: Hounsfield Unit
LAD: Left anterior descending coronary artery
MAE: Mean absolute error
ME: Mean error
MHD: Mean heart dose
NTCP: Normal tissue complication probability
OAR: Organ at risk
ROI: Region of interest
TPS: Treatment planning system
VMAT: Volumetric modulated arc therapy
XVI: X-ray volumetric imaging
